# Tumor-specific antibody cocktail treatment suppresses colorectal tumor growth in mice

**DOI:** 10.1007/s00262-026-04337-8

**Published:** 2026-02-25

**Authors:** Girja S. Shukla, Stephanie C. Pero, Yujing Sun, Linda Mei, Ramiro Barrantes-Reynolds, Matthew R. Fournier, Margaret E. Ackerman, David N. Krag

**Affiliations:** 1 Department of Surgery, Larner College of Medicine, University of Vermont, 89 Beaumont Ave, Burlington, VT 05405 USA; 2 Vermont Integrative Genomics Resource DNA Facility, University of Vermont, Burlington, VT USA; 3 Thayer School of Engineering, Dartmouth College, Hanover, NH USA; 4 Moonshot Antibodies, Inc., Shelburne, VT USA

**Keywords:** Colorectal cancer, Polyclonal antibodies, Antibody cocktail, Immunotherapy, Personalized medicine

## Abstract

**Background:**

Colorectal cancer (CRC) remains a leading cause of cancer-related mortality, with advanced-stage disease frequently marked by treatment resistance and recurrence. Tumor heterogeneity, driven by the accumulation of somatic mutations, undermines the efficacy of conventional therapies and limits the long-term success of targeted agents. There is an urgent need for new therapeutic strategies that can exploit, rather than be constrained by, this heterogeneity.

**Methods:**

We developed a personalized immunotherapeutic pipeline in the syngeneic CT26 murine model of CRC. Briefly, whole exome sequencing identified mutated surface proteins (MSPs) unique to these cells. Of these MSPs, we selected 10 for the generation of MSP-specific polyclonal antibodies (pAbs). These pAbs were tested for specificity and peptide binding to peptides via ELISA, tumor tissue by immunofluorescence, and tumor cells by flow cytometry. Therapeutic efficacy was evaluated in vivo using CT26 tumor-bearing mice treated with the pAb cocktail alone or in combination with anti-PD-1 immune checkpoint blockade. To assess clinical relevance, we analyzed The Cancer Genome Atlas (TCGA) whole exome sequencing data from 100 human CRC patients for MSP prevalence and inter-patient variability.

**Results:**

The 10-pAb oligoclonal antibody cocktail preparations exhibited additive, high-affinity, tumor-specific binding with minimal reactivity to healthy tissues. In vivo, this pAb cocktail significantly suppressed tumor growth and, when combined with PD-1 blockade, prolonged median survival to over 90 days in treated mice compared to less than 25 days in controls. Whole exome sequence data revealed that the majority of human CRC tumors harbored 10 or more MSPs, with minimal overlap between individuals, highlighting the feasibility and necessity of personalized antibody-based therapies.

**Conclusion:**

Our findings establish a proof-of-concept for individualized, mutation-guided antibody therapies, supporting further development of this approach to improve outcomes in patients with advanced CRC.

**Supplementary Information:**

The online version contains supplementary material available at 10.1007/s00262-026-04337-8.

## Introduction

Despite its prominence as the third most common cancer globally and its increasing prevalence among younger individuals, treatment options for colorectal cancer (CRC) remain limited, with no curative options for patients with advanced disease [1, 2]. Even when advanced CRC patients experience promising initial responses to treatment, recurrence is common, contributing to eventual treatment failure and death [3, 4]. Therapeutic resistance and tumor recurrence are driven in large part by the tendency of tumor cells to accumulate mutations and the consequent heterogeneity of malignant cell populations [5], allowing a subset of cells to persist even when therapies markedly reduce overall tumor burden. Despite an apparent clinical response, treatment-resistant tumors ultimately emerge, prompting a need for alternative interventions. Conventional therapies tend to be either largely nonspecific (e.g., chemotherapy and radiotherapy) or to engage specific targets (e.g., tyrosine kinase inhibitors and monoclonal antibodies [mAbs]), and offer benefits to only a subset of patients owing to this inherent intra- and inter-patient heterogeneity, even when deployed in combination. To increase the number of patients who achieve a durable therapeutic response and to prolong the duration of this response, new therapies are thus needed that leverage this tumor-specific heterogeneity as a resource, rather than suffering it as a restriction.

Antibodies have revolutionized the oncology space in recent decades, with key therapeutic breakthroughs stemming from the production of both tumor antigen-specific therapeutic antibodies and immune checkpoint inhibitors (ICIs) [2, 6]. While efforts to treat CRC with both ICIs and growth factor-specific mAbs have shown promise [7], curative outcomes are lacking, and only a subset of patients respond favorably [2, 8]. Owing to their stochastic biogenesis, antibodies offer unmatched potential as a means of tailoring therapeutic solutions to a heterogeneous disease, with estimates of up to one quadrillion (10^15^) members in the naïve human antibody repertoire [9]. A therapeutic pipeline that applies the specificity and diversity of antibodies to simultaneously target multiple distinct mutated proteins across heterogeneous tumor cell populations may better enable tumor control. Targeting multiple different mutated targets increases overall antibody binding to cancer cells without increasing binding to normal cells. We have previously demonstrated the feasibility of producing tumor-specific polyclonal antibody (pAb) cocktails in triple-negative breast cancer, melanoma, and other tumors together with the ability of these antibodies to control tumor growth in mouse models [10–12]. Despite growing interest in the personalized treatment of CRC [13, 14], no similar approach has yet been published in this cancer type, highlighting a new opportunity for therapeutic development with the potential to prolong patient survival and improve quality of life.

In this study, using the murine CT26 model of CRC, we outline a therapeutic pipeline in which tumor-specific mutated cell surface proteins (MSPs) are identified using a bioinformatics approach. We then generate pAbs targeting a subset of these MSPs and demonstrate their tumor-specific binding and ability to prolong the survival of tumor-bearing mice when administered as an oligoclonal antibody cocktail. We additionally highlight the prevalence of MSPs in CRC patients. Together, our results emphasize the promise of personalized antibody treatment as a means of leveraging the inherent heterogeneity present in CRC to more effectively control this devastating disease.

## Results

### Production of pAbs targeting 10 mutated peptides expressed by CT26 cells

**Fig. 1:**
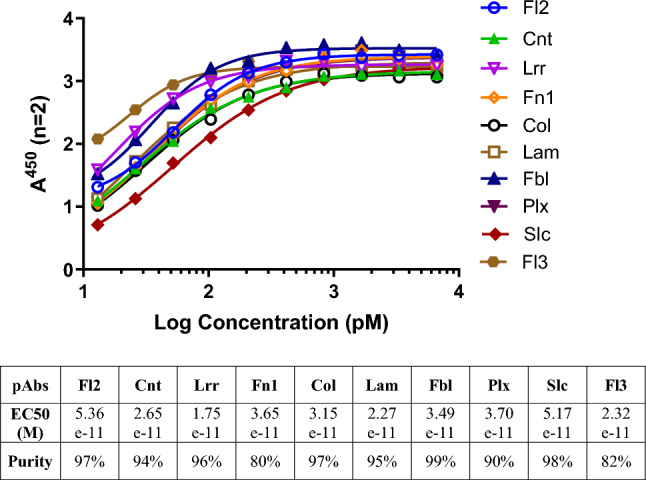
**A** Binding curves for rabbit polyclonal antibodies raised against selected mutated peptides. Each plotted absorbance value represents an average of duplicate (≤ 5%) wells of serially diluted (two-fold) samples of individual rabbit MSP-specific antibodies. **B** EC50 values and purities of affinity-purified rabbit polyclonal antibodies raised against selected mutated CT26 tumor peptides

**Fig. 2:**
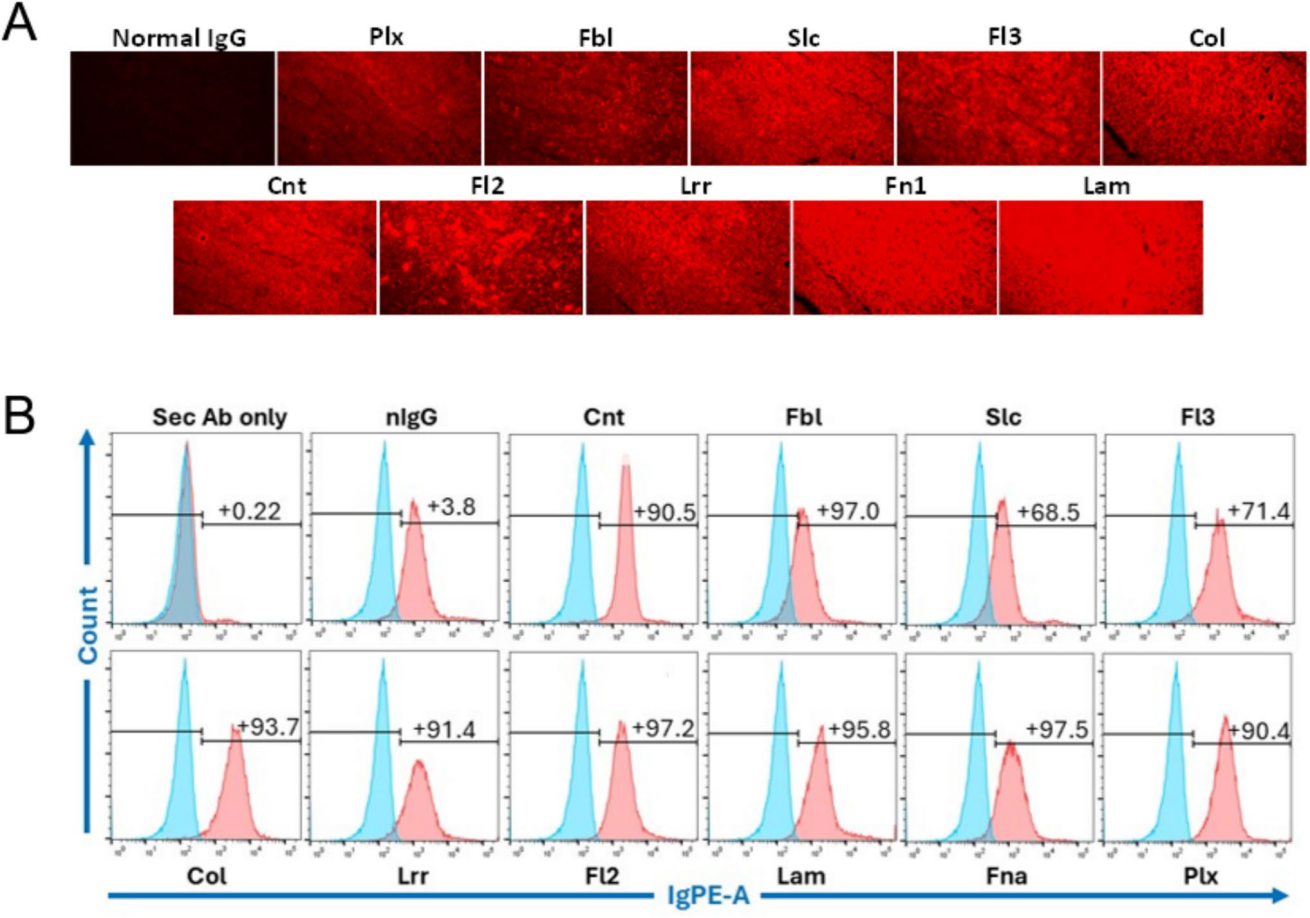
**A** Binding of the 10 selected pAbs and control normal rabbit IgG (nIgG), raised against mutated CT26 proteins, and normal IgG to CT26 tumor tissue sections prepared from tumor-bearing BALB/c mice. Antibodies were tested at a fixed 1 µg/mL concentration. **B** Flow cytometry analyses of binding of the 10 selected pAbs to murine CT26 tumor cells, with normal IgG as an isotype control. Blue indicates the unstained control

To select candidate MSPs as targets, we began by comparing whole exome sequencing data for the CT26 colon tumor cell line to the wild-type BALB/c mouse reference genome. This tumor cell model was selected as it is a syngeneic model, thus allowing for the evaluation of pAb treatment efficacy in the context of an intact host immune system. Of the 1,354 nonsynonymous mutations identified in translated regions in these cells, 153 (11.2%) were in plasma membrane proteins, 76 (5.6%) were in cell junction proteins, and 35 (2.6%) were in extracellular matrix proteins. We then selected 10 distinct surface-associated mutated proteins as targets for antibody-based treatment without taking their functions into account. We generated short 11-mer peptides harboring the identified mutations for each of these 10 MSPs (Table 1), and these peptides were used to vaccinate rabbits in order to generate pAbs targeting these MSPs. Antibody preparations were prepared at > 80% purity, and ELISAs confirmed that all 10 pAb preparations bound to their cognate target antigens with EC_50_ values ranging from 1.75 × 10^−11^ to 5.36 × 10^−11^ M (Fig. 1). Their ability to bind to CT26 tumor cells was further confirmed through immunofluorescence staining using tissue sections prepared from CT26 tumor-bearing BALB/c mice (Fig. 2A), and through flow cytometry demonstrated specific binding not observed for isotype control antibodies (Fig. 2B). Together, these data confirm the predicted surface localization of the 10 chosen MSPs in CT26 tumor cells and underscore the feasibility of generating a series of pAbs capable of binding to tumor-specific mutated proteins.

**Table 1:** Selected peptides derived from the mutated CT26 cell surface proteins used for pAb preparation

Peptide ID	Mutated sequence	Wild-type sequence
Flrt2 (Fl2)	FSIVR**T**SLSHPPPD	FSIVR*N*SLSHPPPD
Cntnap4 (Cnt)	RVRNTHSENA**L**TGV	RVRNTHSENA*H*TGV
Lrrtm2 (Lrr)	ISS**P**SYHVGDKEIP	ISS*S*SYHVGDKEIP
Fn1 (Fn1)	SVVALHDDME**N**QPL	SVVALHDDME*S*QPL
Col8a1 (Col)	GPP**E**IPGPKGEPGL	GPP*G*IPGPKGEPGL
Lamb2 (Lam)	HTMGDVRRAE**E**LLQ	HTMGDVRRAE*Q*LLQ
Fbln5 (Fbl)	VMTRPIKGPR**N**IQL	VMTRPIKGPR*D*IQL
Plxna1 (Plx)	ELKPSSPL**T**LKGRN	ELKPSSPL*I*LKGRN
Slc12a9 (Slc)	FLT**H**PAFSEPAEGT	FLT*D*PAFSEPAEGT
Flrt3 (Fl3)	NQINNVGI**S**SDLKN	NQINNVGI*P*SDLKN

Peptide IDs are represented by the gene names corresponding to the target mutated proteins. The abbreviated gene names used in the present publication are given in parentheses. The mutated amino acid residues are presented in bold font, with the corresponding wild-type amino acids in italics

### Anti-MSP pAbs specifically bind to CT26 tumors in an additive manner

**Fig. 3:**
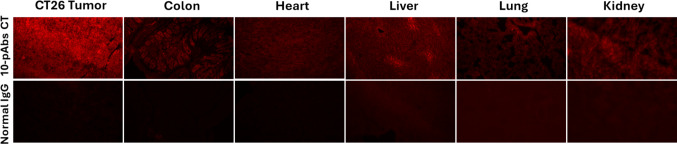
Binding of a cocktail (CT) of 10 tumor-binding pAbs and normal IgG to CT26 mouse tumor and certain normal mouse organ sections. 10-pAb CT = 0.5 µg/mL of 10-Ab cocktail (containing 0.05 µg/mL individual pAbs); Normal IgG = 0.5 µg/mL normal rabbit IgG

**Fig. 4:**
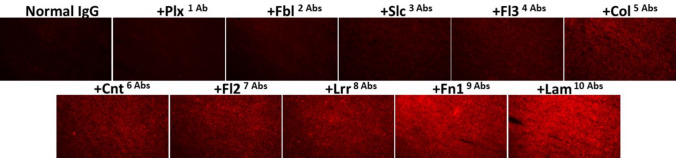
Fluorescent microscopy images of CT26 tumor sections showing increasing binding following the addition of each of the indicated individual pAbs (0.05 µg/mL). Normal rabbit IgG (Normal IgG, 0.5 µg/mL) was used as a negative control

**Fig. 5:**
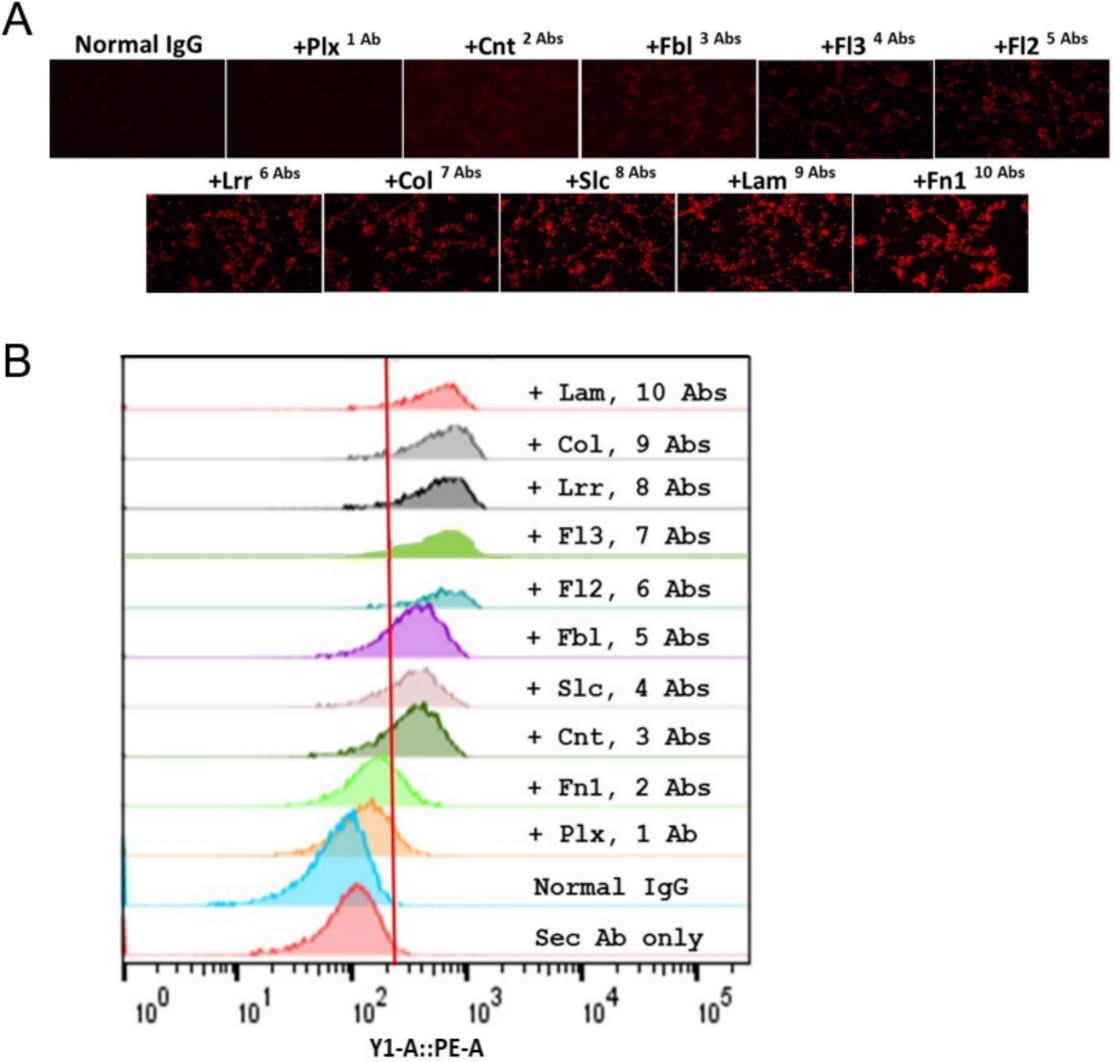
**A** Fluorescence microscopy of cultured CT26 cells show increasing binding following the addition of each selected individual pAb (0.2 µg/mL). Normal rabbit IgG (Normal IgG, 2 µg/mL) was used as a negative control. **B** A compendium of histogram plots from flow cytometry studies to show increasing cell surface binding following addition of each selected individual pAb (0.025 µg/mL). Normal rabbit IgG (Normal IgG, 0.25 µg/mL) and secondary Ab (Sec Ab only) were used as negative controls. The red vertical line shows the 95th percentile binding of the negative controls

Before testing the performance of these pAbs in a therapeutic context, we first sought to confirm that their cumulative binding is specific to tumor cells, given that any high-affinity binding to healthy tissues would contribute to the potential for undesirable therapeutic outcomes. To that end, a cocktail containing equal concentrations of all 10 pAb preparations was generated and used for the immunofluorescent staining of a range of major organs and tissues from BALB/c mice. Only weak binding was observed in any of these healthy tissues, in stark contrast to the strong binding to CT26 tumor sections (Fig. 3). Strikingly, these antibodies exhibited additive binding to CT26 tumors such that the intensity of the immunofluorescent signal when probed with a secondary antibody rose with the sequential addition of each additional anti-MSP antibody to the cocktail used to probe these sections (Fig. 4). Similar additive binding dynamics were also observed via flow cytometry when used to probe cultured CT26 cells (Fig. 5, Supplementary Fig. 1). These results are consistent with the fact that these pAbs target 10 different tumor antigens, potentially allowing all 10 to bind to specific tumor cells or to heterogeneous populations of cells expressing only a subset of these antigens.

### Anti-MSP pAbs suppress CT26 tumor growth and prolong survival

**Fig. 6:**
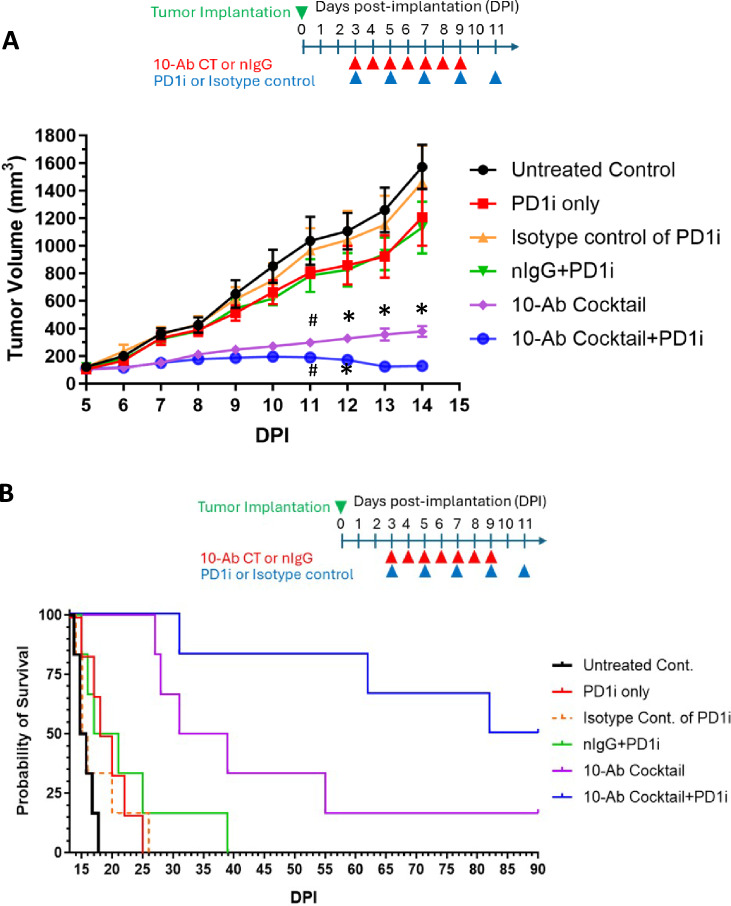
**A**, **B** Effects of treatment of CT26 tumor-bearing mice with a cocktail of 10 antibodies against CT26 colon tumor-derived mutated peptides on tumor weights (**A**) and murine survival (**B**) Significantly different (* = *P* ≤ 0.0001; # = *P* < 0.0003) tumor growth was observed in comparison with Untreated, PD1i, and normal IgG (nIgG) control groups, as determined by Tukey's multiple comparisons test. Data represent mean ± SEM, *n* = 6/group. DPI = Days post-implantation. PD1i, anti-PD-1 treatment

To test the therapeutic utility of an oligoclonal antibody cocktail consisting of these 10 pAbs, CT26 tumor-bearing BALB/c mice were established. Beginning 3 days post-implantation, animals were subcutaneously administered daily injections of the 10-pAb cocktail (or IgG control) alone or in combination with anti-PD-1 (injected intraperitoneally every other day), which was applied to boost antitumor immunity. Anti-PD-1 ICI treatment was included in this experimental design in light of strong evidence supporting the ability of combined ICI treatment to contribute to better immunotherapy outcomes [15–17]. While PD-1 inhibition (PD1i) alone failed to impact tumor growth, the 10-pAb cocktail significantly slowed the growth of target tumors, and combination 10-pAb + PD1i treatment led to the near-total cessation of tumor growth during the treatment period (Fig. 6A, Supplementary Table 1), without any change in body weight (Supplementary Fig. 2). Even after treatment had been discontinued, this combination regimen was associated with profound benefits, prolonging the median survival of treated mice to 86 days as compared to 15.5–19 days in the control groups without 10-Ab cocktail (Fig. 6B, Supplementary Table 2). Applying this anti-MSP antibody cocktail together with immune checkpoint blockade thus holds promise as a new therapeutic modality to prolong survival of individuals with colon cancer.

### Human colon cancer is a feasible target for therapeutic anti-MSP antibody generation

**Fig. 7:**
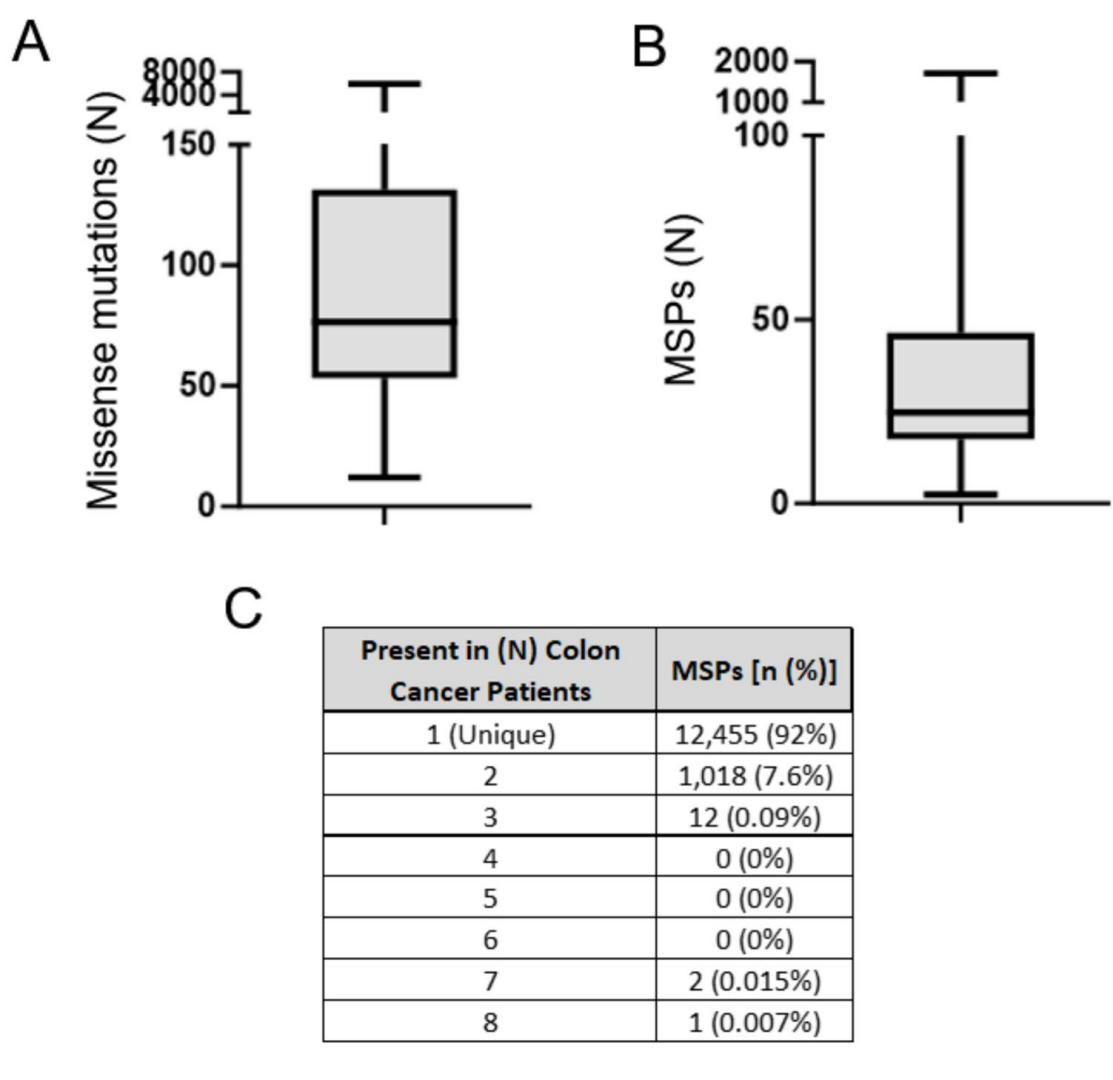
Mutational frequencies and MSP overlap among colon cancer patients in the TCGA database. (A, B Total missense mutations (**A**) and mutated cell surface proteins (MSPs) (**B**) identified in colon cancer patients included in the TCGA database. (**C**) The frequencies of MSP identification among colon cancer patients in the TCGA database. The number in the right column reflects the number (%) of MSPs shared by the number of colon cancer patients from the TCGA database shown in the right column, among 100 total patients included in this analysis

Given the promising efficacy of anti-MSP-targeted antibody treatment in colon tumor-bearing mice and the pressing unmet need in the treatment of advanced colon cancer in humans, we sought to determine the potential amenability of colon cancer patients to this therapeutic strategy. To that end, we leveraged The Cancer Genome Atlas, analyzing available genomic data from 100 colon cancer patients included in this dataset. Comparisons of the available genomic sequencing data from the tumors of these patients and corresponding normal tissues revealed a median of 76.5 (IQR: 5,958, Min: 12; Max: 5,970) missense mutations per patient and a median of 24.75 (IQR: 1,712; Min 2.5; Max 1,714) MSPs per patient (Fig. 7A, B). Based on these results, 96% of colon cancer patients harbor at least 10 MSPs, highlighting the potential feasibility of developing oligoclonal antibody cocktails targeting at least 10 MSPs per patient. Strikingly, despite the high number of total missense mutations identified in this patient cohort, very few of these mutations were shared between patients. Indeed, just 12 MSPs (0.088%; 12/13,488 total unique mutations) were shared by 3 or more patients, and none were shared by more than 8 of these 100 patients (Fig. 7C).

## Discussion

The present study introduces a novel, personalized immunotherapeutic strategy targeting tumor-specific MSPs in CRC, demonstrating its feasibility and therapeutic potential in a murine model. By leveraging the inherent heterogeneity of tumor-specific mutations, our oligoclonal antibody cocktail approach seeks to turn the long-standing clinical challenge posed by inter- and intra-tumoral mutational diversity into a therapeutic advantage. This approach, in which multiple antibodies are generated against patient-specific surface-expressed mutations, not only circumvents the limitations of conventional mAb therapies that lack patient specificity but also opens a new avenue for individualized cancer treatment.

We selected 10 MSPs in the murine CT26 CRC model using whole exome sequencing and a custom bioinformatic pipeline. The subsequent generation of pAbs against short peptides containing each of these mutated sequences resulted in highly specific, high-affinity antibody preparations. All 10 pAbs demonstrated robust and specific binding to CT26 tumor cells with negligible off-target recognition of healthy mouse tissues. Notably, their combined use in an oligoclonal cocktail produced an additive binding pattern, both in tumor tissue sections and in flow cytometric analyses of cultured cells. This suggests that these antibodies recognize distinct epitopes on the surfaces of heterogeneous tumor cell subpopulations, supporting the central hypothesis that targeting multiple MSPs with antibodies can provide broader tumor coverage than traditional monoclonal therapy. Moreover, such oligoclonal antibody-based treatment strategies offer patient-specific design and the ability to avoid the HLA-restricted nature of patient responses that limit the use of peptide vaccines [13].

Therapeutically, the administration of this oligoclonal antibody cocktail significantly inhibited tumor growth in vivo and conferred a marked survival benefit to treated animals. When combined with PD-1 immune checkpoint blockade, the effect was even more pronounced, resulting in near-complete arrest of tumor progression during the treatment period and prolonged survival post-treatment. These data highlight the synergistic potential of combining tumor-targeted antibodies with ICIs, consistent with prior studies demonstrating the value of pairing antigen-targeting therapies with immunomodulatory and other agents to enhance antitumor immunity [15–17].

**Fig. 8:**
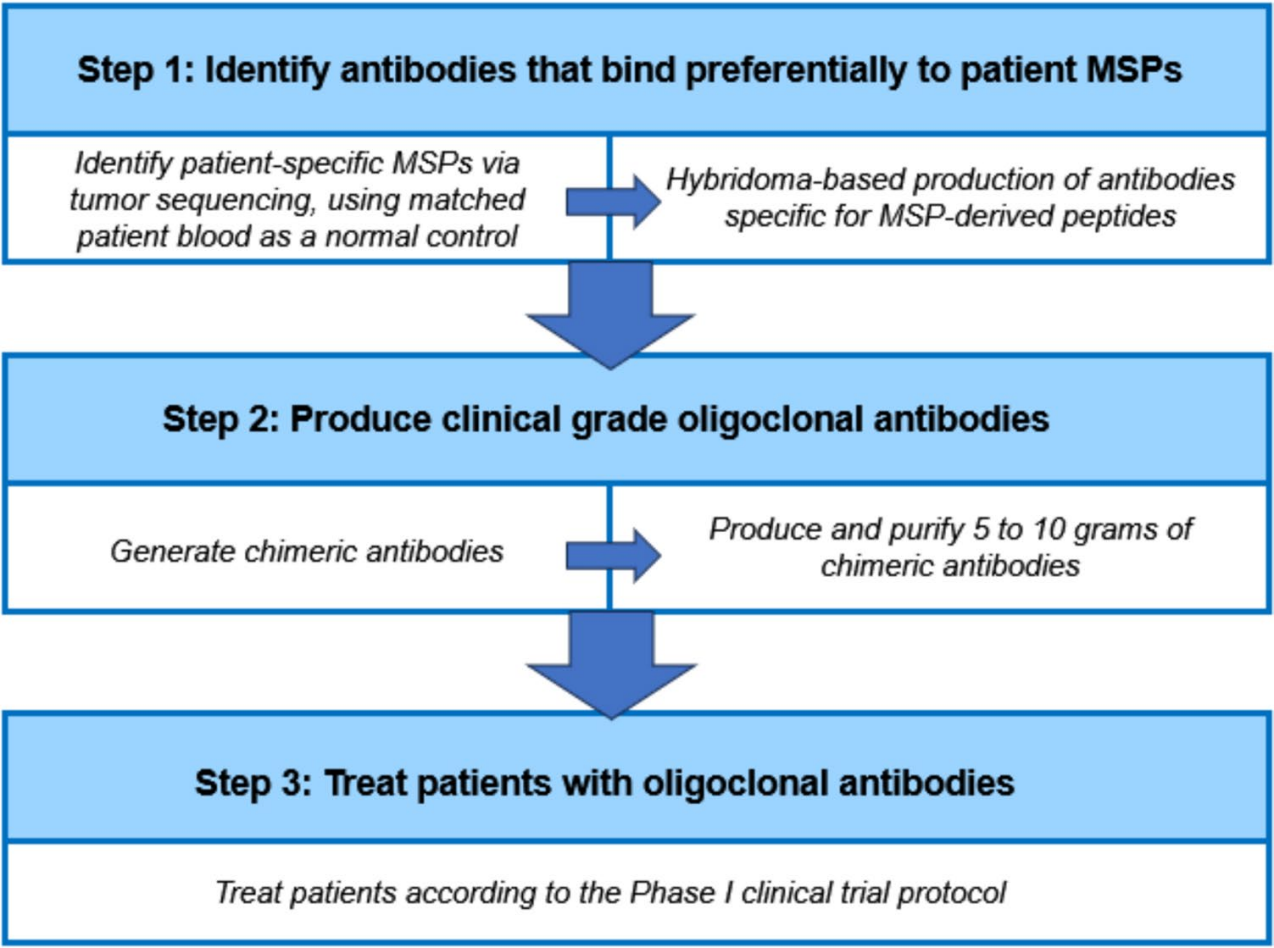
Workflow for the production of personalized MSP-specific oligoclonal antibodies and patient treatment in a clinical trial

One of the most compelling aspects of this study is the demonstration that the therapeutic concept is not limited to the murine model. While the 10 MSPs targeted in the CT26 model system are not relevant to human disease, as the underlying mutations are random and thus tumor- and species-specific, bioinformatic analysis of TCGA data from 100 human CRC patients revealed that the majority harbor a sufficient number of patient-specific MSPs to justify individualized antibody cocktail design. While the median number of MSPs per patient was substantial, inter-patient overlap was minimal, such that only ~ 0.1% of MSPs were shared by more than three individuals, and none were shared by more than eight. As such, while colon tumors tend to harbor high numbers of surface mutations, these mutations are highly patient-specific, emphasizing the lack of suitable targets for more traditional mass-produced therapeutic antibodies. These findings therefore underscore the promise of and need for personalized MSP-targeting antibody cocktails individually prepared for each patient as a means of more effectively eliminating these tumors and improving patient outcomes (Fig. 8). Because these individual mutations are extremely rare and are not likely to contribute to cancer growth, exploring the relationship between individual MSP-associated mutated epitopes and CRC patient survival outcomes is not feasible, nor is it likely to have any direct impact on the efficacy of this exogenous antibody treatment approach.

The therapeutic strategy presented here stands in contrast to conventional approaches that have long sought "universal" tumor antigens as therapeutic targets [18], and have ultimately focused on targeting some of the most common tumor-associated antigens. While major targetable proteins identified to date offer manufacturing and regulatory advantages for antibody production, their clinical utility is inherently limited by tumor heterogeneity [5, 19]. In contrast, personalized MSP-specific Abs addresses this challenge directly by tailoring therapy to the unique mutational landscape of each tumor. This approach also dovetails with broader trends in precision oncology, including the use of tumor neoantigen vaccines and adoptive T cell therapies, both of which rely on patient-specific tumor profiling [13, 14]. Importantly, unlike T cell-based strategies, antibody therapies can be rapidly produced, scaled as needed, and administered without the complexities of cell harvesting, ex vivo expansion, or reinfusion.

There are, however, important limitations and challenges that must be acknowledged. First, although the murine CT26 model provides a useful proof-of-concept evidence as a syngeneic model system suitable, further studies will be needed to validate the efficacy and safety of personalized oligoclonal antibody cocktail treatment more broadly in CRC. Our team has successfully implemented this pAb cocktail-based treatment strategy in other tumor types [10–12, 20], suggesting that this approach is flexible and that its efficacy is not restricted to a particular cell line or cancer type, although it is important to note that, as MSPs are tumor type-specific, new pAbs need to be generated for each experimental model. Second, the generation of high-affinity, specific antibodies for each patient’s unique set of MSPs remains a complex process, particularly within the tight timeframes typically required for therapeutic decision-making in oncology. We are actively working to optimize a therapeutic pipeline comprising sequencing, peptide synthesis, and recombinant antibody production strategies to help address these challenges as we begin a phase 1 clinical trial testing the feasibility of this approach in humans.

Another important consideration is the immune context of the host. Our study demonstrates enhanced tumor control when the anti-MSP pAb cocktail is combined with PD-1 blockade, suggesting that the effectiveness of the antibody treatment is at least partially dependent on host immune responses. This is consistent with an Fc-mediated antibody-dependent cellular cytotoxicity (ADCC)- and/or antibody-dependent cellular phagocytosis (ADCP)-mediated mechanism of action [16, 21]. Future work will be needed to elucidate the specific immune mechanisms through which these MSP-targeting antibodies exert their antitumor effects, including their interactions with natural killer (NK) cells, macrophages, and complement pathways. Engineering Fc regions to enhance ADCC or complement activation could further augment therapeutic efficacy [16, 22]. However, given the inherent complexity of the tumor immune microenvironment found within solid tumors [23], systematic analyses may be essential to clarify the contributions of specific immune cells to the efficacy of this therapeutic strategy and to guide the appropriate selection of ICIs or other combination therapies.

Lastly, while we focused here on MSPs, this strategy could be extended to include other classes of tumor-specific antigens, such as splice variants, fusion proteins, or post-translationally modified peptides uniquely expressed on cancer cells. Broadening the antigenic landscape in this way could further increase tumor coverage and reduce the likelihood of immune escape, provided the targets are accessible to antibody-based targeting. It is important to note that the MSPs chosen for antibody-based targeting in this study were selected to avoid any known oncogenic proteins. While these targets will be relevant in future clinical studies, we excluded them here to minimize any potential functional impacts of antibody treatment on the target proteins beyond their ability to mediate ADCC, underscoring an important avenue for future study.

In conclusion, this study presents a compelling preclinical proof-of-concept for a personalized oligoclonal antibody cocktail-based strategy targeting tumor-specific MSPs in CRC. By capitalizing on the very heterogeneity that undermines current therapies, we show that it is possible to mount a potent and specific immune attack on diverse tumor cell populations. To explore the clinical feasibility and safety of this approach, we have initiated a Phase I clinical trial enrolling stage IV patients with CRC and other cancers (NCT06674538). Through this trial and the insights gained through this study, we hope to extend new individually tailored treatment options to meaningfully prolong survival while spurring a paradigm shift by introducing a viable alternative to conventional treatment approaches.

## Materials and methods

### Chemicals and reagents

Normal rabbit IgG (pH 7.4, protein A-purified, preservative-free, in PBS) was obtained from Sino Biological, Inc. (Wayne, PA). The rat anti-mouse PD-1 antibody (clone RMP1–14, InVivoMAb anti-mouse PD-1) and its isotype control (rat IgG2a, clone 2A3, InVivoMAb IgG2a isotype control) were purchased from Bio X Cell (West Lebanon, NH). These antibodies were supplied in PBS (pH 7.0) and were suitable for in vivo mouse treatments. Goat anti-rabbit IgG conjugated to Alexa Fluor^TM^ 568 and cross-adsorbed goat anti-mouse IgG (H + L) conjugated to horseradish peroxidase (HRP) were obtained from Life Technologies (Carlsbad, CA). All other reagents used were of molecular biology or high purity grade.

### Tumor cell culture

CT26 murine colorectal carcinoma cells (ATCC CRL-2638), were acquired from the American Type Culture Collection (ATCC, Manassas, VA). Cells were cultured in RPMI-1640 (ACC 30–2001) containing 10% fetal bovine serum (ATCC 30–2020) and maintained until ~ 70% confluency. Cells were confirmed to be free of mycoplasma contamination. Cells were detached using Trypsin–EDTA (ATCC), washed, and resuspended in PBS at the desired concentrations for mouse inoculation, following ATCC protocols.

### Selection of CT26 mutated peptides and production of rabbit pAbs

Whole exome sequencing data from CT26 cells [24] were analyzed to identify missense mutations in surface-expressed proteins. Ten MSPs were selected based on the extracellular location of the mutated residues as determined using UniProt [25]. Functional roles of the proteins were not considered during selection. For each mutation, an immunogenic 11-mer peptide containing the mutated residue was synthesized, with a C-terminal cysteine added to facilitate conjugation to keyhole limpet hemocyanin (KLH). GenScript (Piscataway, NJ) produced and affinity-purified rabbit pAbs specific to these peptides. Antibodies were supplied in preservative-free PBS. The selection of the antibodies to be included in the oligoclonal cocktail mixture was based on binding to the CT26 cells and tumor tissue.

### Titration of antibodies against mutated peptides

Affinity-purified rabbit pAbs were titrated using ELISAs to estimate EC_50_ values, as previously described [20]. MaxiSorp^TM^ 96-well plates (Nunc, NY, USA) were coated with individual mutant peptides in carbonate buffer (pH 9.6). After blocking with 1% casein in TBS (Thermo Fisher Scientific, MA, USA), serial twofold dilutions of each pAb were added. Following incubation and washing with TBS containing 0.1% Tween 20, HRP-conjugated anti-rabbit IgG (GenScript) was applied. Binding was visualized using TMB substrate (GenScript), and absorbance was recorded at 450 nm every 2 min over 10 min using a Synergy HT plate reader (BioTek Instruments). EC50 values were calculated using GraphPad Prism (GraphPad Software, CA, USA).

### Immunofluorescence microscopy

Immunofluorescence studies were performed as described previously [11]. Freshly cultured CT26 cells and frozen tissue sections from CT26 tumors and normal mouse organs were fixed with paraformaldehyde. Samples were blocked with Image-iT^TM^ FX Signal Enhancer (Thermo Fisher Scientific), then incubated for 1 h at room temperature with either individual MSP-specific pAbs, a cocktail of 10 pAbs, or control rabbit IgG. Alexa Fluor^TM^ 568-conjugated goat anti-rabbit IgG was used for detection. Slides were mounted with Dako Fluorescent Mounting Medium (Agilent, CA, USA) and visualized using a Nikon TE2000-U inverted fluorescence microscope (Nikon Corp., Kanagawa, Japan) with excitation/emission wavelengths of 579/603 nm.

### pAb cocktail preparation

IgG concentrations of the affinity-purified pAbs were measured using a NanoDrop spectrophotometer (Thermo Fisher Scientific). All 10 pAbs demonstrated specific binding to CT26 tumor tissue, with no detectable binding to normal mouse tissues. These pAbs were pooled in equal mass proportions to generate a final cocktail concentration of 2 mg/mL in US Pharmacopeia (USP)-grade PBS.

### Flow cytometry

Flow cytometry was performed to assess the binding of anti-mutated peptide pAbs to the surface of CT26 cells, following a previously published method [26]. CT26 cells were incubated in PBS containing 1% BSA with either individual pAbs, the 10-antibody cocktail, or control rabbit IgG. After blocking with donkey serum, cells were stained using PE-conjugated donkey anti-rabbit IgG (BioLegend, San Diego, CA). A Live/Dead (blue) viability stain (Thermo Fisher Scientific) was used to exclude dead cells. Cells were fixed with paraformaldehyde and analyzed on a BD LSR II flow cytometer using BD FACSDiva^TM^ v8 software. Data were processed and gated using FlowJo^TM^ v10.1 (FlowJo LLC, Ashland, OR), with appropriate isotype and unstained controls.

### Animal tumor model

A syngeneic CT26 colon carcinoma model was established in immunocompetent BALB/c mice. CT26 is an undifferentiated colon carcinoma cell line derived from BALB/c mice and is known for its aggressive growth characteristics. Female BALB/c mice aged 6–7 weeks (± 3 days) were purchased from The Jackson Laboratory (Bar Harbor, ME, USA). Mice were acclimated for one week in the animal facility prior to experimentation. Animals were housed in individually ventilated cages supplied with filtered air and maintained under standard conditions, including a 12-h light/dark cycle. Food and water were provided ad libitum.

### Animal treatments

Experiment 1: This experiment aimed to identify the optimal number of CT26 cells required to produce tumors reaching approximately 2000 mm^3^ within 15–20 days post-inoculation. Mice were randomly assigned to three groups (n = 6/group), and subcutaneously injected in the shaved left flank with 5 × 10^5^, 1 × 10^6^, or 1.5 × 10^6^ CT26 cells suspended in phosphate-buffered saline (PBS). Tumor growth was monitored by measuring tumor dimensions at designated time intervals.

Experiment 2: This experiment assessed the effects of a rabbit MSP-targeting pAb cocktail, administered alone or in combination with PD-1 inhibitor (PD1i), on CT26 tumor growth and mouse survival. All mice were subcutaneously inoculated with 1 × 10^6^ CT26 cells. Animals were then randomized into six treatment groups (n = 6/group): Group 1—Untreated control: No treatment administered; Group 2—PD1i only: Intraperitoneal (i.p.) injections of 0.2 mg PD1i per mouse on days 3, 5, 7, 9, and 11 post-inoculation (DPI); Group 3—PD1i isotype control: i.p. injections of 0.2 mg isotype control antibody on the same schedule as Group 2; Group 4—Normal rabbit IgG + PD1i: Subcutaneous (s.c.) injections of 0.2 mg normal rabbit IgG at the tumor base on DPI 3–9, plus i.p. injections of 0.2 mg PD1i on DPI 3, 5, 7, 9, and 11; Group 5—Antibody cocktail only: S.c. injections of 0.2 mg of a cocktail containing 10 rabbit polyclonal antibodies against mutated peptides at the tumor base on DPI 3–9; and Group 6—Antibody cocktail + PD1i: Combined treatment with the antibody cocktail (s.c. injections as in Group 5) and PD1i (i.p. injections as in Group 2).

### Animal data analyses

Mice were monitored throughout the study for signs of adverse effects related to treatment. Body weights were recorded twice weekly. Additional observations included food and water intake, general behavior, and appearance to assess animal welfare.

Tumor growth was tracked from 7 DPI using electronic calipers. Tumor volume was calculated using the formula: *V* = (*w*^2^ × *l*)/2, where w = tumor width and *l* = tumor length. Survival time was determined based on either natural death or euthanasia due to tumor burden reaching ≥ 2000 mm^3^ or signs of distress (e.g., dehydration, impaired mobility, and cachexia), in accordance with the Institutional Animal Care and Use Committee (IACUC) guidelines.

### CRC patient missense mutation analyses

The human patient somatic mutation information and variant calls were obtained from the open-access collection of The Cancer Genome Atlas Project (TCGA; https://www.cancer.gov/tcga) with available matching tumor and normal blood data. To identify the subcellular localization of each missense mutation, the UniProtID mapping service was used [25, 27]. First, the correct UniProtID for each gene was identified to ensure the correct isoform was considered. Entries with no UniProtID were eliminated as data on subcellular localization could not be obtained. For each UniProtID, annotated subcellular location information was obtained from Uniprot with the uniprotR package (https://cran.r-project.org/web/packages/UniprotR/citation.html). Given the variant position in the gene, the exact subcellular location of the variant was identified, including whether the variant was in the extracellular domain or located on a secreted protein. Programming was performed using R [28], and reports were generated using RStudio [29].

### Statistical analyses

Statistical analyses were performed using GraphPad Prism (GraphPad Software, San Diego, CA) and Microsoft Excel. Tumor growth differences among groups were evaluated using two-way analysis of variance (ANOVA), followed by Tukey’s multiple comparison test. Survival data were presented as Kaplan–Meier plots. Differences between survival curves were analyzed using the Log-rank (Mantel-Cox) test, with median survival times reported. Confidence intervals (CIs) for median survival and hazard ratios (Mantel–Haenszel) were calculated at the 95% confidence level. A p value < 0.05 was considered statistically significant.

## Supplementary Information

Below is the link to the electronic supplementary material.

Supplementary file1 (DOCX 236 kb)

## Data Availability

The datasets generated during and/or analyzed during the current study are available from the corresponding author on reasonable request.
