# Supplementary material for: Tumor-specific antibody cocktail treatment suppresses colorectal tumor growth in mice
Source: Cancer Immunol Immunother. 2026 Feb 25;75(3):87. doi: 10.1007/s00262-026-04337-8 (PMC12936289; doi:10.1007/s00262-026-04337-8)
Supplement: Supplementary file 1 — Supplementary file1 (DOCX 236 kb) [file 262_2026_4337_MOESM1_ESM.docx]

**Supplemental Materials**

**Supplementary Figure 1.** The gating strategy for flow cytometry analyses of the cell-surface binding of a cocktail of (A) 10 tumor-binding pAbs and (B) normal IgG to CT26 tumor cells. 10-Ab Cocktail = 0.5 µg/mL 10-Ab cocktail (containing 0.05 µg/mL of each of 10 individual pAbs); Normal IgG = 0.5 µg/mL normal rabbit IgG.

**Supplementary Figure 2.** Body weight remains unchanged over the course of pAb cocktail treatment in CT26 tumor-bearing mice. These data correspond to the experiment shown in Figure 6.

**Supplementary Table 1.** Significance of tumor weight curve differences (Log-rank, Mantel-Cox test) in the different treatment groups, in comparison to the respective controls.

| **2-Way ANOVA** | SS | DF | MS | F (DFn, DFd) | P-value |
| --- | --- | --- | --- | --- | --- |
| Interaction | 10058770 | 45 | 223528 | F (45, 300) = 4.017 | P<0.0001 |
| Time | 26498945 | 9 | 2944327 | F (9, 300) = 52.92 | P<0.0001 |
| Treatment | 18698347 | 5 | 3739669 | F (5, 300) = 67.21 | P<0.0001 |

| **Supplementary Table 2.** Significance of survival curve differences (Log-rank, Mantel-Cox test) in the different treatment groups, in comparison to the respective controls. |
| --- |

| Group | Chi Square | DF | P-value | Median survival  (d) | Log-rank hazard ratio | 95% CI |
| --- | --- | --- | --- | --- | --- | --- |
| All curves | 35.81 | 5 | <0.0001 |  |  |  |
| Untreated control vs  10-Ab Cocktail+PD1i | 10.29 | 1 | 0.0013 | 15.5 vs  86.0 | 6.430 | 1.384 to 29.87 |
| Untreated control vs  10-Ab Cocktail | 12.16 | 1 | 0.0005 | 15.5 vs 35.0 | 4.982 | 1.118 to 22.20 |
| nIgG+PD1i vs  10-Ab Cocktail+PD1i | 9.99 | 1 | 0.0016 | 19.0 vs 86.0 | 6.009 | 1.328 to 27.19 |
|  |  |  |  |  |  |  |
| PD1i vs  10-Ab Cocktail+PD1i | 10.09 | 1 | 0.0005 | 19.0 vs 86.0 | 6.651 | 1.412 to 31.32 |
